# A quantitative association study of *SLC25A12 *and restricted repetitive behavior traits in autism spectrum disorders

**DOI:** 10.1186/2040-2392-2-8

**Published:** 2011-05-24

**Authors:** Soo-Jeong Kim, Raquel M Silva, Cindi G Flores, Suma Jacob, Stephen Guter, Gregory Valcante, Annette M Zaytoun, Edwin H Cook, Judith A Badner

**Affiliations:** 1 Department of Psychiatry, University of Florida, Gainesville, FL, USA; 2 Department of Psychiatry, University of Illinois at Chicago, Chicago, IL, USA; 3 Department of Psychiatry, University of Chicago, Chicago, IL, USA

## Abstract

**Background:**

*SLC25A12 *was previously identified by a linkage-directed association analysis in autism. In this study, we investigated the relationship between three *SLC25A12 *single nucleotide polymorphisms (SNPs) (rs2056202, rs908670 and rs2292813) and restricted repetitive behavior (RRB) traits in autism spectrum disorders (ASDs), based on a positive correlation between the G allele of rs2056202 and an RRB subdomain score on the Autism Diagnostic Interview-Revised (ADI-R).

**Methods:**

We used the Repetitive Behavior Scale-Revised (RBS-R) as a quantitative RRB measure, and conducted linear regression analyses for individual SNPs and a previously identified haplotype (rs2056202-rs2292813). We examined associations in our University of Illinois at Chicago-University of Florida (UIC-UF) sample (179 unrelated individuals with an ASD), and then attempted to replicate our findings in the Simons Simplex Collection (SSC) sample (720 ASD families).

**Results:**

In the UIC-UF sample, three RBS-R scores (ritualistic, sameness, sum) had positive associations with the A allele of rs2292813 (p = 0.006-0.012) and with the rs2056202-rs2292813 haplotype (omnibus test, p = 0.025-0.040). The SSC sample had positive associations between the A allele of rs2056202 and four RBS-R scores (stereotyped, sameness, restricted, sum) (p = 0.006-0.010), between the A allele of rs908670 and three RBS-R scores (stereotyped, self-injurious, sum) (p = 0.003-0.015), and between the rs2056202-rs2292813 haplotype and six RBS-R scores (stereotyped, self-injurious, compulsive, sameness, restricted, sum)(omnibus test, p = 0.002-0.028). Taken together, the A alleles of rs2056202 and rs2292813 were consistently and positively associated with RRB traits in both the UIC-UF and SSC samples, but the most significant SNP with phenotype association varied in each dataset.

**Conclusions:**

This study confirmed an association between *SLC25A12 *and RRB traits in ASDs, but the direction of the association was different from that in the initial study. This could be due to the examined *SLC25A12 *SNPs being in linkage disequilibrium with another risk allele, and/or genetic/phenotypic heterogeneity of the ASD samples across studies.

## Background

Autism spectrum disorders (ASDs) are characterized by qualitative impairments in reciprocal social interaction and communication, and by the presence of restricted repetitive behavior (RRB) [1]. ASDs are highly heritable complex genetic disorders with rare variants, oligogenic inheritance, and interactions between susceptibility alleles [2-8]. The heterogeneity of ASDs makes it difficult to identify risk alleles, but also supports the validity of a model that requires more than one genetic variant to contribute to the full syndrome of autism [9,10]. *SLC25A12 *(solute carrier family 25 member 12; OMIM *603667) on chromosome 2q24 encodes aralar, a mitochondrial aspartate-glutamate carrier isoform 1 (AGC1) protein. *SLC25A12 *spans about 110 kb. *SLC25A12 *was initially identified as an autism-susceptibility gene through a linkage-directed association study and replication [11-14]. For instance, two independent groups reported overtransmission of the G alleles of two *SLC25A12 *SNPs in intron 3 (rs2056202) and intron 16 (rs2292813) in autism families [12,13]. Other groups also reported overtransmission of the G allele of either rs2056202 [11] or rs2292813 [14], or undertransmission of the A-A haplotype of rs2056202-rs2292813 [14] in autism families. Most recently, the G allele of rs908670, another *SLC25A12 *SNP in intron 8, showed an evidence for overtransmission in a genome-wide association study (GWAS) by the Autism Genome Project (AGP) Consortium (p = 0.0006 in combined AGP, Autism Genetic Resource Exchange (AGRE), and Study on Addiction: Genetics and Environment (SAGE) samples) [15]. However, not all studies have found evidence for association between *SLC25A12 *and autism [16-19]. This conflicting data may be explained by differences in phenotypic characteristics and/or genetic heterogeneity across study samples.

Interestingly, Silverman *et al*. (2008) examined the correlation between *SLC25A12 *and phenotypic data obtained from the Autism Diagnostic Interview-Revised (ADI-R), and found a positive correlation between the G allele of rs2056202 and an RRB-related subdomain, the 'routines and rituals' score [20]. This subdomain consists of two ADI-R items: 'verbal rituals' and 'compulsion/ritualistic behavior'. However, apart from the Silverman study, no other studies have examined the association between *SLC25A12 *and quantitative RRB traits.

In the present study, we hypothesized that *SLC25A12 *may confer risk for quantitative RRB traits in ASDs. We tested this hypothesis by examining associations between three *SLC25A12 *SNPs (rs2056202, rs908670 and rs2292813) and the Repetitive Behavior Scale-Revised (RBS-R), a quantitative measure of RRB. We examined associations first in our University of Illinois at Chicago-University of Florida (UIC-UF) sample (179 unrelated people with an ASD), and then attempted to replicate our findings in the Simons Simplex Collection (SSC) sample (720 ASD families). Because the SSC sample has parental genotype data available for these SNPs, we also examined transmission disequilibrium using family-based association tests.

## Methods

### Subjects and assessment

#### UIC-UF sample

This study was approved by the UIC and UF Institutional review boards (IRBs). All participants were provided with a description of the study before informed consent was obtained. The study participants (179 unrelated people with an ASD) were recruited mainly from two geographical regions (UIC sample from the Chicago, Illinois area; UF sample from north central Florida).

All UIC participants were assessed with the ADI-R [21] and the Autism Diagnostic Observation Schedule (ADOS) [22]. For this report, we required all subjects to meet ASD or autism classification on both ADI-R and ADOS, along with a best-estimate diagnosis of an ASD (i.e., autistic disorder, Asperger disorder, or pervasive developmental disorder-not otherwise specified) by the criteria of the *Diagnostic and Statistical Manual of Mental Disorders*, Fourth Edition, Text Revision (DSM-IV-TR) [1]. We excluded probands with an insufficient DNA sample for genotyping, and/or who lacked RBS-R data. In total, 88 probands (75 male, 13 female; mean age 8.3 ± 4.8 years) were identified as meeting the above criteria as of the data freeze on 1 December 2009, coinciding with data submission to National Database for Autism Research. In this group, 64.8% of participants were white; 6.8% were on concurrent psychotropic medications (these subjects were excluded from the neurochemical analyses of Autism Centers of Excellence (ACE) but were included for this report); and 72.7% were classified as 'strictly defined autism', as they met the autism classification on both ADI-R and ADOS. There were seven missing RBS-R data points (completion rate of 99.8%). These missing data points were treated as 'missing' for both affected subscale and sum scores.

For the UF sample, the inclusion criteria were chronological age between 6 and 18 years, clinical diagnosis of an ASD, sufficient DNA sample available for genotyping, and an absence of any specific genetic diagnosis. Because the UF sample was recruited primarily from a mail survey study, an independent validation of the clinical diagnoses was not conducted, therefore, we used the Social Communication Questionnaire (SCQ) [23] and excluded anyone with an SCQ total score less than 15 in this report, as a validation study had suggested that the SCQ discriminated well between patients with ASD and those with non-ASD, with a sensitivity of 0.85 and a specificity of 0.75 [24]. The UF sample consisted of 91 subjects with an ASD (75 male, 16 female; mean age 10.8 ± 3.6 years); 74.7% were white and 63.7% were on concurrent psychotropic medications. There were no missing RBS-R data points in the UF sample.

#### SSC sample

The phenotype and genotype data were obtained through the Simons Foundation Autism Research Initiative (SFARI) Base [25] with approval from the UF IRB and SSC. In total, 737 probands had available genotype data released on 27 July 2010; we excluded seventeen probands for this report; three probands who were twins of designated probands, and fourteen probands whose phenotypic data were not yet available from the SFARI collection version 10 released on 1 November 2010. Therefore, the SSC sample used in this report included 720 children (age 6.9 ± 2.8 years) who met the criteria for ASD or autism on both the ADI-R and ADOS (563 with 'strictly defined autism') and their biological parents (720 trio families). There were 620 male and 100 female children, and 81% of the children were white. There were 56 missing RBS-R data points (completion rate 99.8%), which were treated as 'missing' for both affected subscale and sum scores.

#### RRB assessment

We used the RBS-R as our primary RRB measure. The RBS-R is an empirically derived, standardized and psychometrically sound rating scale, targeting a variety of abnormal repetitive behaviors [26,27]. The RBS-R includes forty-three individual items grouped into six empirically derived subscales: stereotyped behavior, self-injurious behavior, compulsive behavior, ritualistic behavior, sameness behavior, restricted behavior, and a sum score. A recent factor analysis by Lam and Aman (2007) produced a five-factor solution; stereotyped behavior, self-injurious behavior, compulsive behavior, ritualistic/sameness behavior and restricted interests [28].

#### SNP genotyping

Three *SLC25A12 *SNPs (rs2056202, rs908670, rs2292813) were selected for this study, because rs2056202 and rs2292813 were the original two SNPs associated with ASDs, and rs908670 was the most strongly associated SNP within *SLC25A12 *in the AGP GWAS [15]. The SNPs were genotyped using a commercial assay (TaqMan^® ^SNP Genotyping Assay; Applied Biosystems, Foster City, CA, USA) for the UIC-UF sample. For the SSC sample, the genotype data for these SNPs were available from the Illumina^® ^1M/1Mduo Genechip data (https://sfari.org/sfari-base).

### Statistical analyses

The associations between quantitative RRB measures (RBS-R subscale and sum scores) and specific SNPs or haplotypes were tested using linear regression analyses, as implemented in PLINK (http://pngu.mgh.harvard.edu/~purcell/plink/). The '--hap-omnibus' option was used to jointly estimate all haplotype effects at that position. The potential confounding factors including age, gender, population (white versus non-white) and recruitment site (UIC versus UF for the UIC-UF sample), were treated as covariates. For the SSC sample, we used the DFAM and QFAM tests implemented in PLINK to examine the family-based association for qualitative traits (ASDs) and quantitative traits (RRB). In addition, for the SSC sample only, we added full scale IQ (FSIQ) into the linear regression model as an additional covariate to examine the effect of FSIQ. The '--mperm 10,000' option was used to generate a single-point *p *value (EMP1) to correct for non-normal trait distributions, and a permutation *p *value to correct for multiple SNPs/haplotypes (but not multiple phenotypes) in this study. Furthermore, we transformed the mean and standard deviation of RBS-R subscale and sum scores to '0' and '1' to obtain a 'standardized' β (a regression coefficient) allowing β to be comparable across different samples and phenotypes. The criterion for significance was set at a permutation *p *value of <0.05. We also denoted any finding with a permutation *p *value of >0.05 but <0.1 as a trend in this report.

The IBM^® ^SPSS^® ^Statistics software (version 19; SPSS Inc., Chicago, IL, USA) was used for descriptive analyses to compare the characteristics across the three samples (UIC versus UF versus SSC), including age, gender, population (white versus non-white), three adaptive behavior domains (communication, daily living, socialization) and composite scores on the Vineland Adaptive Behavior Scale-II (VABS-II) [29], the RBS-R subscales and the Aberrant Behavior Checklist (ABC) [30] factor scores (Table 1).

**Table 1:** Descriptive analyses of sample characteristics across the sites of recruitment^a^

	UIC (n = 88)	UF (n = 91)	SSC (n = 720)	*P *value (χ^2^/ANOVA)^b^	*Post hoc *(Scheffé)
Age in years^a^	8.3 ± 4.8	10.8 ± 3.6	6.9 ± 2.8	< 0.001	UF>>^c^
Gender, M:F	75:13	75:16	620:100	NS	
Race, white:other	57:31	68:23	583:137	0.001	
Communication	76.2 ± 13.9	68.0 ± 17.0	79.4 ± 13.9	< 0.001	SSC>>^d^
Daily living	75.1 ± 12.5	71.6 ± 17.9	78.6 ± 13.8	< 0.001	SSC>>
Socialization	67.5 ± 10.3	62.4 ± 16.0	72.7 ± 12.7	< 0.001	SSC>>
Adaptive behavior composite	71.7 ± 11.0	66.1 ± 14.7	75.1 ± 11.8	< 0.001	SSC>>
RBS-R					
Stereotyped behavior	6.2 ± 4.1	5.4 ± 3.3	4.1 ± 3.2	< 0.001	SSC<<^e^
Self-injurious behavior	3.4 ± 4.8	4.1 ± 4.5	2.0 ± 2.8	< 0.001	SSC<<
Compulsive behavior	6.9 ± 5.5	6.1 ± 5.1	4.1 ± 3.8	< 0.001	SSC<<
Ritualistic behavior	7.2 ± 5.4	6.2 ± 4.3	5.2 ± 3.9	< 0.001	SSC<<
Sameness behavior	11.1 ± 8.0	10.9 ± 7.1	7.8 ± 6.0	< 0.001	SSC<<
Restricted behavior	5.6 ± 3.3	4.8 ± 3.3	3.6 ± 2.7	< 0.001	SSC<<
Sum	37.4 ± 21.8	36.5 ± 19.6	26.9 ± 17.1	< 0.001	SSC<<
ABC					
Irritability	16.0 ± 11.1	16.2 ± 10.2	10.9 ± 8.5	< 0.001	SSC<<
Lethargy	14.0 ± 9.0	13.5 ± 8.6	9.5 ± 6.9	< 0.001	SSC<<
Stereotypy	6.5 ± 4.9	6.8 ± 5.0	4.6 ± 4.0	< 0.001	SSC<<
Hyperactivity	21.0 ± 12.5	21.2 ± 11.3	16.0 ± 10.2	< 0.001	SSC<<
Inappropriate speech	4.6 ± 3.4	4.3 ± 3.1	3.7 ± 2.9	< 0.01	SSC<
Sum	62.1 ± 32.9	62.0 ± 28.5	44.7 ± 24.6	< 0.001	SSC<<

^a^Data are mean ± SD unless otherwise stated.

^b^χ^2^/ANOVA: frequency data (gender, race) were analyzed by χ^2 ^statistics, whereas the remaining scale data were analyzed by ANOVA statistics,

^c^UF>>: higher mean age in the UF sample compared with those of UIC-ACE or SSC.

^d^SSC>>: higher mean scores in the SSC sample compared with those of UIC-ACE or UF.

^e^SSC<<: lower mean scores in the SSC sample compared with those of UIC-ACE or UF.

The effect of each individual covariate on RBS-R was examined using linear regression analyses in PLINK, while controlling for the effects of the other covariates and tested SNPs. The significance was set at an uncorrected p value of <0.05. The mean RBS-R subscale and sum scores for individual genotypes of the three SNPs were calculated using the PLINK option of '--qt-mean' (Table 2). Hardy-Weinberg equilibrium (HWE) and Mendelian errors were examined using the PLINK options of '--hwe' and '--me'. Quanto software (version 1.1; University of Southern California, CA, USA; quanto@icarus2.usc.edu) was used for a power calculation assuming an additive mode of inheritance and type 1 error rate of 0.05 [31].

**Table 2:** The individual SNP genotypes versus RBS-R subscale and sum mean scores

UIC-UF sample (n = 179 probands)									
SNP	rs2056202	rs2056202	rs2056202	rs908670	rs908670	rs908670	rs2292813	rs2292813	rs2292813

Genotype^a^	A/A	A/G	G/G	G/G	G/A	A/A	A/A	A/G	G/G

N^b^	6	66	106	15	76	86	3	52	124

Stereotyped behavior	5.5 ± 3.4	6.6 ± 4	5.3 ± 3.5	7.1 ± 4.6	5.5 ± 3.7	5.8 ± 3.6	4.3 ± 3.2	6.9 ± 3.9	5.4 ± 3.6
Self-injurious behavior	2.2 ± 1.9	4.8 ± 5.6	3.1 ± 3.9	4.2 ± 6.4	3.1 ± 4.2	4.3 ± 4.8	2.3 ± 1.5	5.1 ± 5.7	3.2 ± 4.2
Compulsive behavior	6.5 ± 6.2	6.7 ± 5.3	6.4 ± 5.3	9.5 ± 7.9	6 ± 4.7	6.2 ± 5	4.3 ± 4	7.4 ± 5.6	6.1 ± 5.2
Ritualistic behavior	4.8 ± 3.7	*8.3 ± 5.5^c^*	5.9 ± 4.2	6.1 ± 4.8	6.4 ± 4.8	7 ± 5	3.3 ± 3.5	**8.8 ± 5.3^d^**	5.9 ± 4.4
Sameness behavior	10.2 ± 8.3	12.9 ± 8.6	9.9 ± 6.6	10.5 ± 7.5	10.4 ± 7.1	11.5 ± 7.8	10 ± 11.4	**13.8 ± 8.7^d^**	9.9 ± 6.7
Restricted behavior	3.7 ± 3.6	5.8 ± 3.4	4.9 ± 3.1	5.1 ± 3.6	5.1 ± 3.5	5.4 ± 3.1	3.3 ± 3.2	6.3 ± 3.4	4.8 ± 3.2
Sum	32.8 ± 21.9	45.8 ± 26.5	35.4 ± 19.6	42.6 ± 24.5	36 ± 21.8	41.2 ± 23.2	27.7 ± 22.7	48.5 ± 26^d^	35.5 ± 20.2

SSC sample (n = 720 probands)									

SNP	rs2056202	rs2056202	rs2056202	rs908670	rs908670	rs908670	rs2292813	rs2292813	rs2292813

Genotype^a^	A/A	A/G	G/G	G/G	G/A	A/A	A/A	A/G	G/G

N^b^	21	176	523	67	284	367	10	127	583

Stereotyped behavior	**6 ± 3.6^d^**	4.4 ± 3.2	4 ± 3.1	3.3 ± 2.8	4.1 ± 3	**4.4 ± 3.3^d^**	5.6 ± 3.8	4.2 ± 2.9	4.1 ± 3.2
Self-injurious behavior	**3.8 ± 4.5^d^**	2.1 ± 2.9	1.9 ± 2.6	1.4 ± 2	1.8 ± 2.4	**2.3 ± 3.1^d^**	2.8 ± 3	2 ± 2.6	2 ± 2.8
Compulsive behavior	5.5 ± 4.8	4.3 ± 3.9	4 ± 3.7	3.3 ± 3	4.1 ± 4	4.3 ± 3.8	5.7 ± 5.1	3.9 ± 3.6	4.1 ± 3.8
Ritualistic behavior	6.4 ± 4.9	5.6 ± 4	5.1 ± 3.8	4.8 ± 3.7	5.3 ± 3.7	5.3 ± 4	6.1 ± 5.5	5.4 ± 3.8	5.2 ± 3.8
Sameness behavior	**10.1 ± 7.5^d^**	8.6 ± 6.2	7.5 ± 5.9	6.9 ± 5.8	7.9 ± 6.1	8 ± 6.1	10 ± 8.7	8.5 ± 6.1	7.6 ± 6
Restricted behavior	**5.2 ± 3.1^d^**	3.9 ± 3.1	3.4 ± 2.6	2.9 ± 2.5	3.6 ± 2.7	3.6 ± 2.8	*6 ± 2.4^c^*	3.8 ± 3.1	3.5 ± 2.6
Sum	**35.5 ± 21.7^d^**	28.8 ± 18.2	25.9 ± 16.3	22.7 ± 14.9	26.9 ± 16.5	27.8 ± 17.8	36.2 ± 21.2	27.4 ± 17	26.6 ± 17

^a^The SNP genotypes are based on positive strand.

^b^Total number of probands with genotype data available.

Permutation *p *values: ^c^*p *< 0.1; ^d^*p *< 0.05.

### Post hoc analyses

We also conducted analysis of covariance (ANCOVA) to examine the four ADI-R scores highlighted in the Silverman study [20] (i.e., age at phrase speech, overall level of language, circumscribed interests, and routines and rituals) by rs2056202 and rs2292813 genotype groups in both the UIC (n = 88 probands) and SSC (n = 720 probands) samples, using the General Linear Model, as implemented in the IBM^® ^SPSS^® ^Statistics software, with gender and age treated as covariates. These analyses were performed mainly because the direction of associated alleles in our study differed from the Silverman study [20]. Because the 'age at phrase speech' item included non-scale codes (e.g., 994, 996 and 997), we treated these codes as 'missing'. The numbers of non-scale codes were 16 in the UIC sample and 50 in the SSC sample. The three genotype groups were reduced to two (A/+ versus G/G) due to the low frequency of the A/A genotype.

## Results

There were seven missing SNP genotypes [UIC-UF sample: rs2056202 (n = 1), rs908670 (n = 2) and rs2292813 (n = 0); SCC sample: rs2056202 (n = 1), rs2292813 (n = 1) and rs908670 (n = 2)]. The linkage disequilibrium (R^2^) was 0.07 between rs2056202 and rs908670, 0.70 between rs2056202 and rs2292813 and 0.05 between rs908670 and rs2292813. The minor allele frequencies (MAF) were 0.22 (UIC-UF) and 0.15 (SSC) for the A allele of rs2056202, 0.30 (UIC-UF) and 0.29 (SSC) for the G allele of rs908670, and 0.16 (UIC-UF) and 0.10 (SSC) for the A allele of rs2292813. The range of allele frequencies of the A alleles of rs2056202 and rs2292813 have been reported to be 0.09 to 0.20 in previous studies [13,14,16,18,20]. The distributions of the genotypes were consistent with HWE, and the SSC family data were free of Mendelian errors.

Descriptive analyses of sample characteristics (Table 1) showed that the levels of adaptive behaviors were much higher in the SSC sample, followed by the UIC and UF samples. In addition, the SSC participants had lower levels of maladaptive behaviors measured on the RBS-R and ABC, whereas there were no significant differences in the mean scores of RBS-R and ABC between the UIC and UF samples. Interestingly, the mean scores of the RBS-R appeared to be related to the individual SNP genotypes (Table 2); for instance, subjects with A/G genotypes rs2056202 and rs2292813 had higher RBS-R scores than those with A/A or G/G genotypes in the UIC-UF sample. However, there were too few subjects with A/A genotypes of rs2056202 or rs2292813 for a valid interpretation. In the SSC sample, a trend toward reduction in RBS-R subscale and sum scores was seen across three genotype groups (A/A > A/G > G/G).

The linear regression analyses for individual SNPs revealed significant positive associations between the A allele of rs2292813 and three RBS-R scores (ritualistic, sameness, sum; permutation *p *= 0.017, 0.018, 0.034, respectively) in the UIC-UF sample. The SSC sample had significant positive associations between the A allele of rs2056202 and four RBS-R scores (stereotyped, sameness, restricted, sum; permutation *p *= 0.026, 0.021, 0.016, 0.027, respectively) and significant positive associations between the A allele of rs908670 and two RBS-R subscales (stereotyped, self-injurious; permutation *p *= 0.040, 0.009, respectively) (Table 3). For the SSC sample only, FSIQ was added into the linear regression model as a covariate along with age, gender and population (Table 4); the association results remained similar to the results shown in Table 3.

**Table 3:** Linear regression analyses for association between quantitative RRB traits and *SLC25A12 *SNPs after controlling for age, gender and population

Sample	UIC-UF sample (n = 179 probands)			SSC sample (n = 720 probands)		
SNP	rs2056202 (n^a ^= 178)	rs908670 (n = 177)	rs2292813 (n = 179)	rs2056202 (n = 720)	rs908670 (n = 718)	rs2292813 (n = 720)

Risk allele (frequency)	A (0.219)	G (0.299)	A (0.162)	A (0.151)	G (0.291)	A (0.102)

Stereotyped behavior						

β^b^	0.201	0.126	0.262	0.187	-0.138	0.055
Stat^c^	1.501	1.075	1.775	2.591	-2.440	0.639
EMP1^d^	0.138	0.287	0.077	0.008	0.015	0.524
Permutation *p*	0.303	0.563	0.178	**0.026^e^**	**0.040^e^**	0.851

Self-injurious behavior						

β^b^	0.196	-0.072	0.276	0.164	-0.168	0.009
Stat^c^	1.413	-0.591	1.807	2.258	-2.966	0.110
EMP1^d^	0.162	0.552	0.069	0.026	0.003	0.910
Permutation *p*	0.351	0.870	0.168	*0.059^f^*	**0.009^e^**	0.999

Compulsive behavior						

β^b^	0.024	0.199	0.138	0.123	-0.093	0.004
Stat^c^	0.174	1.667	0.901	1.669	-1.621	0.049
EMP1^d^	0.865	0.097	0.375	0.097	0.110	0.960
Permutation *p*	0.996	0.227	0.692	0.224	0.247	1.000

Ritualistic behavior						

β^b^	0.298	-0.096	0.414	0.120	-0.029	0.043
Stat^c^	2.173	-0.797	2.761	1.652	-0.511	0.502
EMP1^d^	0.029	0.420	0.007	0.099	0.617	0.621
Permutation *p*	*0.073^f^*	0.747	**0.017^e^**	0.233	0.915	0.919

Sameness behavior						

β^b^	0.284	-0.081	0.420	0.194	-0.062	0.156
Stat^c^	2.036	-0.678	2.757	2.679	-1.087	1.820
EMP1^d^	0.039	0.497	0.006	0.008	0.281	0.069
Permutation *p*	0.103	0.827	**0.018^e^**	**0.021^e^**	0.556	0.169

Restricted behavior						

β^b^	0.096	-0.047	0.279	0.203	-0.068	0.166
Stat^c^	0.703	-0.391	1.840	2.823	-1.208	1.952
EMP1^d^	0.480	0.691	0.070	0.006	0.230	0.054
Permutation *p*	0.807	0.953	0.160	**0.016^e^**	0.475	0.127

Sum						

β^b^	0.260	-0.046	0.390	0.188	-0.110	0.085
Stat^c^	1.848	-0.379	2.548	2.553	-1.917	0.977
EMP1^d^	0.066	0.701	0.012	0.010	0.052	0.331
Permutation *p*	0.163	0.961	**0.034^e^**	**0.027^e^**	0.131	0.627

^a^Number of subjects with non-missing genotype data available.

^b^Standardized regression coefficient (negative value indicates negative correlation).

^c^Coefficient t-statistics.

^d^Uncorrected single-point *p *value.

Permutation *p *value: ^e^*p *< 0.05; ^f^*p *< 0.1.

**Table 4:** Linear regression analyses for association between quantitative RRB traits and *SLC25A12 *SNPs after controlling for age, gender, population and FSIQ in the SSC sample

Sample	SSC sample (n = 720 probands)		
SNP	rs2056202 (n^a ^= 714)	rs908670 (n = 712)	rs2292813 (n = 714)

Risk allele (frequency)	A (0.151)	G (0.291)	A (0.102)

Stereotyped behavior			

β^b^	0.655	-0.444	0.211
Stat^c^	2.883	-2.499	0.783
EMP1^d^	0.005	0.012	0.441
Permutation *p*	**0.012^e^**	**0.033^e^**	0.763

Self-injurious behavior			

β^b^	0.484	-0.468	0.044
Stat^c^	2.408	-2.990	0.183
EMP1^d^	0.016	0.003	0.857
Permutation *p*	**0.044^e^**	**0.008^e^**	0.995

Compulsive behavior			

β^b^	0.531	-0.356	0.055
Stat^c^	1.915	-1.651	0.169
EMP1^d^	0.057	0.096	0.872
Permutation *P*	0.132	0.230	0.996

Ritualistic behavior			

β^b^	0.491	-0.114	0.183
Stat^c^	1.749	-0.519	0.551
EMP1^d^	0.077	0.606	0.573
Permutation *p*	0.186	0.904	0.889

Sameness behavior			

β^b^	1.222	-0.377	0.975
Stat^c^	2.797	-1.095	1.880
EMP1^d^	0.005	0.280	0.061
Permutation *p*	**0.014^e^**	0.548	0.151

Restricted behavior			

β^b^	0.579	-0.188	0.469
Stat^c^	2.953	-1.223	2.019
EMP1^d^	0.003	0.220	0.042
Permutation *p*	**0.008^e^**	0.462	0.107

Sum			

β^b^	3.485	-1.889	1.615
Stat^c^	2.784	-1.937	1.096
EMP1^d^	0.005	0.057	0.276
Permutation *p*	**0.015^e^**	0.135	0.546

^a^Number of subjects with non-missing genotype data available.

^b^Standardized regression coefficient (negative value indicates negative correlation).

^c^Coefficient t-statistics.

^d^Uncorrected single-point *p *value.

Permutation *p *value: ^e^*P*< 0.05.

The haplotype analyses were conducted for the previously identified haplotype (rs2056202-rs2292813) (Table 5). In the UIC-UF sample, the haplotype omnibus test revealed significant associations with three RBS-R scores (ritualistic, sameness, sum; permutation *p *= 0.028, 0.025, 0.040, respectively). Individual haplotype analyses were consistent with the omnibus test, with positive correlations between the A-A haplotype and three RBS-R scores (ritualistic, sameness, sum; permutation *p *= 0.020, 0.017, 0.029, respectively), and a trend toward negative correlations between the G-G haplotype and ritualistic behavior (permutation *p *= 0.078). In the SSC sample, the haplotype omnibus test showed significant associations with six RBS-R scores (stereotyped, self-injurious, ritualistic, sameness, restricted, sum; permutation *p *= 0.002, 0.003, 0.021, 0.028, 0.019, 0.009, respectively). Individual haplotype analyses revealed positive correlations between the A-G haplotype and four RBS-R scores (stereotyped, self-injurious, compulsive, sum; permutation *p *= 0.002, 0.002, 0.020, 0.012, respectively) and negative associations between the G-G haplotype and four RBS-R scores (stereotyped, sameness, restricted, sum; permutation *p *= 0.022, 0.023, 0.014, 0.031, respectively). As a comparison, three-SNP-haplotype analyses (rs2056202-rs908670-rs2292813) were also conducted (Table 6), which showed similar trends to those from the two-SNP-haplotype analyses shown in Table 5.

**Table 5:** Haplotype analyses for association between quantitative RRB traits and rs2056202-rs2292813 haplotype

	UIC-UF sample (n = 179 probands)				SSC sample (n = 720 probands)			
Haplotype (frequency)	A-A (0.163)	A-G (0.056)	G-G (0.781)	Omnibus	A-A (0.107)	A-G (0.045)	G-G (0.849)	Omnibus

Stereotyped behavior								

β^a^	0.271	-0.062	-0.201	N/A	0.054	0.408	-0.186	N/A
Stat^b^	3.400	0.078	2.250	3.380	0.394	11.700	6.650	12.500
EMP1^c^	0.062	0.787	0.134	0.184	0.531	0.000	0.009	0.002
Permutation p	0.146	0.955	0.285	0.184	0.802	**0.002^d^**	**0.022^d^**	**0.002^d^**

Self-injurious behavior								

β^a^	0.285	-0.102	-0.196	N/A	0.010	0.431	-0.165	N/A
Stat^b^	3.500	0.203	2.000	3.500	0.014	13.000	5.140	13.100
EMP1^c^	0.064	0.644	0.155	0.173	0.914	0.001	0.022	0.003
Permutation p	0.139	0.887	0.319	0.173	0.993	**0.002^d^**	*0.054^e^*	**0.003^d^**

Compulsive behavior								

β^a^	0.132	-0.222	-0.024	N/A	0.005	0.324	-0.123	N/A
Stat^b^	0.734	0.962	0.030	1.440	0.003	7.220	2.810	7.300
EMP1^c^	0.389	0.329	0.860	0.487	0.957	0.007	0.095	0.021
Permutation p	0.659	0.581	0.983	0.487	0.998	**0.020^d^**	0.209	**0.021^d^**

Ritualistic behavior								

β^a^	0.406	-0.096	-0.298	N/A	0.042	0.242	-0.119	N/A
Stat^b^	7.340	0.181	4.720	7.300	0.237	4.080	2.680	4.500
EMP1^c^	0.007	0.666	0.032	0.028	0.626	0.043	0.104	0.110
Permutation p	**0.020^d^**	0.903	*0.078^e^*	**0.028^d^**	0.878	0.104	0.222	0.110

Sameness behavior								

β^a^	0.420	-0.156	-0.284	N/A	0.155	0.223	-0.193	N/A
Stat^b^	7.550	0.465	4.140	7.550	3.270	3.470	7.100	7.350
EMP1^c^	0.008	0.510	0.043	0.025	0.073	0.065	0.010	0.028
Permutation p	**0.017^d^**	0.780	0.103	**0.025^d^**	0.164	0.148	**0.023^d^**	**0.028^d^**

Restricted behavior								

β^a^	0.293	-0.383	-0.096	N/A	0.166	0.228	-0.202	N/A
Stat^b^	3.840	3.000	0.493	5.840	3.790	3.680	7.930	8.160
EMP1^c^	0.049	0.088	0.482	0.054	0.053	0.056	0.006	0.019
Permutation p	0.118	0.194	0.759	*0.054^e^*	0.121	0.128	**0.014^d^**	**0.019^d^**

Sum								

β^a^	0.392	-0.171	-0.260	N/A	0.084	0.349	-0.188	N/A
Stat^b^	6.530	0.541	3.410	6.580	0.941	8.130	6.470	9.630
EMP1^c^	0.011	0.465	0.069	0.040	0.325	0.006	0.012	0.009
Permutation p	**0.029^d^**	0.735	0.154	**0.040^d^**	0.577	**0.012^d^**	**0.031^d^**	**0.009^d^**

^a^Standardized regression coefficient (negative value indicates negative correlation).

^b^Wald test was used to compare tested haplotype with the remaining haplotypes.

^c^Uncorrected single-point *P *value.

Permutation *p *value: ^d^*p *< 0.05; ^e^*p *< 0.1. N/A: not applicable

**Table 6:** Haplotype analyses for association between quantitative RRB traits and rs2056202-rs908670-rs2292813 haplotype

	UIC-UF sample (n = 179 probands)					SSC sample (n = 720 probands)				
Haplotype (frequency)	ATA (0.162)	GCG (0.299)	ATG (0.056)	GTG (0.483)	Omnibus	ATA (0.107)	GCG (0.281)	ATG (0.045)	GTG (0.568)	Omnibus

Stereotyped behavior										

β^b^	0.261	0.126	-0.061	-0.212	N/A	0.171	-0.441	1.300	0.064	N/A
Stat^c^	3.150	1.160	0.075	4.300	5.470	0.394	6.010	11.700	0.154	16.100
EMP1^d^	0.078	0.271	0.782	0.036	0.138	0.524	0.013	0.001	0.700	0.001
Permutation p	0.236	0.656	0.991	0.130	0.138	0.907	**0.049^d^**	**0.003^d^**	0.972	**0.001^d^**

Self-injurious behavior										

β^b^	0.276	-0.069	-0.102	-0.058	N/A	0.028	-0.464	1.190	0.151	N/A
Stat^c^	3.260	0.327	0.201	0.285	3.290	0.014	8.730	13.000	1.120	19.600
EMP1^d^	0.076	0.561	0.649	0.596	0.351	0.907	0.005	0.001	0.289	0.001
Permutation p	0.228	0.927	0.964	0.941	0.351	0.999	**0.015^d^**	**0.002^d^**	0.665	**0.001^d^**

Compulsive behavior										

β^b^	0.138	0.200	-0.222	-0.175	N/A	0.016	-0.352	1.230	0.058	N/A
Stat^c^	0.811	2.760	0.963	2.700	4.640	0.002	2.640	7.220	0.087	8.940
EMP1^d^	0.362	0.099	0.328	0.103	0.202	0.959	0.099	0.007	0.765	0.030
Permutation p	0.772	0.300	0.726	0.310	0.202	1.000	0.307	**0.026^d^**	0.989	**0.030^d^**

Ritualistic behavior										

β^b^	0.414	-0.095	-0.096	-0.105	N/A	0.167	-0.114	0.936	-0.139	N/A
Stat^c^	7.620	0.627	0.181	0.974	7.590	0.252	0.269	4.110	0.489	4.560
EMP1^d^	0.006	0.427	0.675	0.324	0.054	0.619	0.599	0.046	0.489	0.211
Permutation p	**0.024^d^**	0.836	0.969	0.719	*0.054^e^*	0.952	0.947	0.142	0.879	0.211

Sameness behavior										

β^b^	0.420	-0.080	-0.156	-0.105	N/A	0.945	-0.378	1.350	-0.280	N/A
Stat^c^	7.600	0.436	0.468	0.942	7.570	3.310	1.200	3.490	0.810	7.630
EMP1^d^	0.007	0.513	0.502	0.336	0.060	0.071	0.263	0.059	0.368	0.056
Permutation p	**0.027^d^**	0.899	0.890	0.736	*0.060^e^*	0.212	0.645	0.192	0.769	*0.056^e^*

Restricted behavior										

β^b^	0.279	-0.046	-0.383	-0.015	N/A	0.454	-0.186	0.624	-0.125	N/A
Stat^c^	3.380	0.143	2.920	0.019	5.430	3.810	1.460	3.700	0.801	8.470
EMP1^d^	0.066	0.705	0.085	0.893	0.147	0.051	0.225	0.055	0.368	0.038
Permutation p	0.213	0.977	0.270	0.999	0.147	0.164	0.563	0.176	0.770	**0.038^d^**

Sum										

β^b^	0.389	-0.045	-0.171	-0.118	N/A	1.450	-1.890	5.960	-0.046	N/A
Stat^c^	6.490	0.137	0.543	1.170	6.520	0.955	3.700	8.150	0.003	11.500
EMP1^d^	0.012	0.706	0.457	0.276	0.094	0.329	0.056	0.006	0.959	0.009
Permutation p	**0.044^d^**	0.981	0.861	0.656	*0.094^e^*	0.723	0.175	**0.019^d^**	1.000	**0.009^d^**

^a^Standardized regression coefficient (negative value indicates negative correlation).

^b^Wald test was used to compare tested haplotype with the remaining haplotypes.

^c^Uncorrected single-point *P *value.

Permutation p value: ^d^*p *< 0.05; ^e^*p *< 0.1. N/A: not applicable.

We also examined transmission disequilibrium (TD) of the three *SLC25A12 *SNPs in the SSC sample using the DFAM test as implemented in PLINK, because previous studies [11-14] suggested overtransmission of the G allele(s) of rs2056202 and/or rs2292813; however, we did not find any evidence of overtransmission of either allele (Table 7). In previous studies [11-14], the transmission rates of the G alleles of rs2056202 and rs2292813 were estimated at approximately 59 to 65% for rs2056202 and 57 to 65% for rs2292813. We examined if the SSC sample had enough power to detect TD, assuming an additive model for a qualitative trait and type 1 error rate of 0.05, using Quanto software. The SSC sample (720 trios) had 80% power to detect a locus with a relative risk of 1.4, which roughly translates to a transmission rate of 58%. Hence, the SSC sample had adequate power to detect an effect size similar to that detected in previous studies. On the other hand, the QFAM test for the quantitative RRB traits revealed positive associations between the A alleles of all three SNPs and the RBS-R scores (Table 8), which was consistent with linear regression analyses results.

**Table 7:** DFAM analyses for family-based association tests for ASD and *SLC25A12 *SNPs in the SSC sample (n = 720 trio families)

SNP	A1^a^	A2^b^	Obs^c^	Exp^d^	χ^2^	*P*
rs2056202	A	G	190	190	0	1
rs908670	G	A	352	337	1.531	0.216
rs2292813	A	G	134	140.5	0.619	0.431

^a^Minor allele.

^b^Major allele.

^c^Observed frequency.

^d^Expected frequency.

**Table 8:** QFAM analyses for family-based association tests for quantitative RRB traits and *SLC25A12 *SNPs in the SSC sample (n = 720 trio families)

	rs2056202 (n^a ^= 720)	rs908670 (n = 718)	rs2292813 (n = 720)
Risk allele (frequency)	A (0.152)	G (0.281)	A (0.107)

Stereotyped behavior			

β^b^	0.208	-0.140	0.087
EMP1^c^	0.004	0.014	0.309
Permutation p	**0.004**^d^	**0.014**^d^	0.316

Self-injurious behavior			

β^b^	0.186	-0.171	0.029
EMP1^c^	0.010	0.003	0.738
Permutation p	**0.016**^d^	**0.001**^d^	0.742

Compulsive behavior			

β^b^	0.131	-0.095	0.012
EMP1^c^	0.070	0.098	0.885
Permutation p	*0.072*^e^	*0.083*^e^	0.883

Ritualistic behavior			

β^b^	0.141	-0.036	0.063
EMP1^c^	0.049	0.532	0.461
Permutation p	*0.053*^e^	0.534	0.471

Sameness behavior			

β^b^	0.197	-0.064	0.154
EMP1^c^	0.006	0.265	0.073
Permutation p	**0.008**^d^	0.270	*0.078*^e^

Restricted behavior			

β^b^	0.233	-0.078	0.200
EMP1^c^	0.001	0.172	0.019
Permutation p	**0.001**^d^	0.162	**0.020**^d^

Sum			

β^b^	0.211	-0.115	0.105
EMP1^c^	0.004	0.046	0.224
Permutation *p*	**0.005**^d^	**0.047**^d^	0.220

^a^Number of subjects with non-missing genotype data available.

^b^Standardized regression coefficient (negative value indicates negative correlation).

^c^Uncorrected single-point P value.

Permutation *p *value: ^d^*p *< 0.05; ^e^*p *< 0.1.

Table 9 shows our *post hoc *analyses, the attempted replication of the Silverman study [20] for the examination of four ADI-R scores by rs2056202 and rs2292813 genotype groups in the samples from UIC (n = 88 probands) and SSC (n = 720 probands). The results from the Silverman study are included in the table for a comparison. Neither UIC nor SSC sample replicated the findings of the Silverman study; however, the SSC sample showed a trend toward more severe 'overall level of language' score (p < 0.05) in the G/G genotype groups of both rs2056202 and rs2292813. We estimated that the Silverman study had a standardized β of 0.42 for 'routines and rituals' and rs2056202 (adjusted mean difference of 0.51 and standard deviation of 1.2). We calculated our study power to see whether our samples had an adequate power to replicate Silverman's finding, using Quanto with an assumption of an additive model of a quantitative locus and type 1 error rate of 0.05. We estimated that the UIC-UF sample could detect a standardized β of 0.43 (R^2 ^= 0.05) and the SSC sample a standardized β of 0.19 (R^2 ^= 0.01), thus, we did have adequate power to replicate Silverman's study.

**Table 9:** Four ADI-R Trait scores highlighted by Silverman [20], grouped by the presence or absence of at least one A allele for rs2056202 and rs2292813.^a,b^

	Age at phrase speech^c^, months	Level of language	Circumscribed interests	Routines and rituals
UIC sample (n = 88 probands)				

rs2056202				

1+A allele (n = 50)	40.0 ± 3.3	0.28 ± 0.09	1.80 ± 0.23	1.44 ± 0.25
G/G (n = 120)	40.5 ± 2.4	0.21 ± 0.07	1.72 ± 0.18	1.81 ± 0.20
Statistics	*F*_(1,70) _= 0.02, *p *= 0.903	*F*_(1,86) _= 0.38, p = 0.542	*F*_(1,86) _= 0.08, *p *= 0.783	*F*_(1,86) _= 1.36, *p *= 0.248

rs2292813				

1+A allele (n = 50)	39.2 ± 3.7	0.27 ± 0.11	1.72 ± 0.26	1.44 ± 0.29
G/G (n = 120)	40.8 ± 2.3	0.23 ± 0.07	1.76 ± 0.16	1.76 ± 0.18
Statistics	*F*_(1,70) _= 0.17, *p *= 0.679	*F*_(1,86) _= 0.10, *p *= 0.753	*F*_(1,86) _= 0.02, *P *= 0.883	*F*_(1,86) _= 0.91, *p *= 0.344

SSC sample (n = 720 probands)				

rs2056202				

1+A allele (n = 50)	37.5 ± 1.4	0.03 ± 0.02	1.84 ± 0.08	1.68 ± 0.10
G/G (n = 120)	38.3 ± 0.9	0.10 ± 0.01	1.87 ± 0.05	1.63 ± 0.06
Statistics	*F*_(1,668) _= 0.29, *p *= 0.590	*F*_(1,718) _= 6.71, ***p *= 0.010^e^**	*F*_(1,718) _= 0.12, *p *= 0.735	*F*_(1,718) _= 0.15, *p *= 0.701

rs2292813				

1+A allele (n = 50)	38.0 ± 1.7	0.03 ± 0.03	1.71 ± 0.10	1.67 ± 0.12
G/G (n = 120)	38.1 ± 0.8	0.09 ± 0.01	1.90 ± 0.05	1.64 ± 0.06
Statistics	*F*_(1,668) _= 0.01, *p *= 0.943	*F*_(1,718) _= 4.42, ***p *= 0.036^e^**	*F*_(1,718) _= 2.97, *p *= 0.085^f^	*F*_(1,718) _= 0.06, *p *= 0.812

Silverman study^d ^(n = 170 probands)				

rs2056202				

1+A allele (n = 50)	45 ± 2	0.90 ± 0.12	1.31 ± 0.11	0.79 ± 0.17
G/G (n = 120)	44 ± 4	0.63 ± 0.08	1.54 ± 1.21	1.30 ± 0.11
Statistics	*F*_(1,166) _= 0.05, *p *= 0.83	*F*_(1,166) _= 3.25, *p *= 0.07^f^	*F*_(1,166) _= 1.36, *p *= 0.25	*F*_(1,166) _= 6.49, ***p *= 0.0117^e^**

^a^Gender and age were used as covariates and estimated marginal means ± standard errorsE are reported.

^b^All *p *values are uncorrected for multiple comparisons.

^c^993-999 codes were treated as missing for Age at phrase speech.

^d^Silverman (2008) study data are shown for comparison.

*P *value: ^e^*p*< 0.05; ^f^*p*< 0.1.

Analyses of the effect of an individual covariate on the RBS-R revealed negative correlations between age and stereotyped behavior (β = -0.004 to -0.005; p < 0.005 to 0.0001), and between female gender and restricted behavior (β = -0.228 to -0.242; p < 0.05). In addition, positive correlations were shown between age and sameness behavior (β = 0.002; p < 0.01), between female gender and self-injurious behavior (β = 0.217; p < 0.05), and between population other than white, and self-injurious, ritualistic, and restricted behaviors (β = -0.188 to 0.329; p < 0.05 to 0.005). Additionally, we found that FSIQ was a significant covariate for several RBS-R subscale scores in the SSC sample, which included stereotyped behavior (p = 0.00001 to 0.00003), self-injurious behavior (p = 0.019 to 0.027), compulsive behavior (p = 0.0002 to 0.0004) and RBS-R sum score (p = 0.0009 to 0.0017).

## Discussion

*SCL25A12 *was implicated in ASD through a linkage-directed association study [12] and more than one independent replication association study [11,13,14], support from a recent GWAS [15], and its role in central nervous system development [32-35]. However, not all association studies between *SLC25A12 *and ASDs have been positive [17-19], indicating the need for further investigation of the basis of this inconsistency.

In the present study, we examined *SLC25A12 *as a quantitative trait locus for RRB in people with ASDs, based on a positive correlation between the G allele of rs2056202 and an ADI-R 'routines and rituals' subdomain score [20]. We initially found evidence for positive associations between the A allele of rs2292813 and RBS-R scores (ritualistic, sameness, sum) and between the A-A haplotype of rs2056202-rs2292813 and the same RBS-R scores in our UIC-UF sample. Although our finding of association between *SLC25A12 *and quantitative RRB traits (ritualistic, sameness behaviors) in the UIC-UF sample appeared to be comparable with the previous association finding [20], the direction of the associated allele was different (the A alleles of rs2056202 and rs2292813 in our samples versus the G allele of rs2056202 in the Silverman study).

This 'flip-flop' phenomenon brought up an important question about whether this study provides a confirmation of an association between *SLC25A12 *and RRB versus a false positive finding [36]. To clarify this issue, we examined the association in a much larger sample consisting of 720 trio families available from the SFARI database. Although the significantly associated SNPs-RRB varied between these two samples (i.e., rs2292813 in the UIC-UF sample, rs2056202/rs908670 in the SSC sample), both datasets showed consistently positive associations with the A alleles of rs2056202 and rs2292813, as evidenced by the positive β values in Table 3 and Table 4. The β values for the A-A and the G-G haplotypes were also consistent across the UIC-UF and SSC samples in the haplotype analyses (positive for the A-A haplotype and negative for the G-G haplotype in Table 5). The A-G haplotype was somewhat puzzling initially, as the A-G haplotype was found to have a negative β in the UIC-UF sample but a positive β in the SSC sample. Interestingly, in the UIC-UF sample, the positively associated 'A' allele of rs2292813 was present only on the A-A haplotype, whereas the negatively associated 'G' allele of rs2292813 was present on both A-G and G-G haplotypes, creating a negative β value for the A-G haplotype. In the SSC sample, by contrast, the more positively associated A allele of rs2056202 was present on the A-A haplotype about 70% of the time, and on the A-G haplotype about 30% of the time, creating a positive β value for the A-G haplotype. Therefore, the individual haplotype associations were consistent with the allelic associations; that is, positive association with the A allele of rs2292813 in the UIC-UF sample, and positive association with the A allele of rs2056202 in the SSC sample. In addition, the significantly associated SNPs and phenotypes may vary between datasets even in a true association [37]. For example, varying patterns of LD across samples could lead to the susceptibility variant to be associated with different variants in different samples. Taken together, these results argue against the probability of a false positive in these (UIC-UF and SSC) samples, despite the direction of the association being different from the Silverman study.

In this study, we also attempted to replicate the Silverman study directly, using the UIC and SSC samples, because it was not clear whether differences in the phenotype used (RBS-R in our study versus ADI-R in the Silverman study) or in the analytic methods (linear regression in our study versus ANCOVA in the Silverman study) might be contributing to the opposite direction of associated allele. Even with the same phenotypes and comparable analytic methods used; however, our samples did not replicate the Silverman findings. This suggests that genetic and phenotypic heterogeneity of ASD samples may at least partly account for the differences across the studies. For instance, we estimated that 51% of the A/+ group, 57% of the G/G group and 55% of the entire Silverman sample had an overall level of language score of 0. These numbers contrast with 82% of the UIC group and 93% of the SSC group having a score of 0 in the overall level of language. In addition, 'overall level of language' would have affected the ADI-R score of 'verbal rituals' and the subdomain score of 'routines and rituals,' which may have influenced the association findings. Furthermore, it is possible that we overestimated our study power based on an estimate of effect size from the Silverman study. This is often referred as 'winner's curse' when the true effect size may have been much smaller than an estimate from the primary study [38]. Another point to consider is that the ADI-R is not as quantitative as the RBS-R. Therefore, scores may have not provided sufficient variability to observe the same association.

Several family-based association studies previously identified the G alleles of rs2056202 or rs2292813 as risk alleles for autism [11-14]. Although it sounds confusing, these results should not be confused with our study result (the A alleles of rs2056202 or rs2292813 associated with RRB), because our study did not examine associations with autism, but with RRB. Although not straightforward, the association is not expected to be the same even if they seem to be related (i.e., RRB and autism), when it is not the same phenotype. In other words, there is variability of RRB in subjects diagnosed with ASDs. If there were not, then it would not be possible to detect an association with degree of RRB within an autism sample. If an allele is associated with increasing RRB within an autism sample, then whether that allele will show an association with autism depends on the distribution of RRB in the sample. The association with autism may be with the same allele, the opposite allele or neither allele in a sample in which RRB tends to be high, low or mixed within the autism sample, respectively.

In this study, we did not find evidence for TD between ASDs and *SLC25A12 *in the SSC sample. Because the SSC sample was estimated to have adequate power to detect a locus with a relative risk of 1.4, this result further emphasizes the genetic heterogeneity of ASD (making the effect size smaller or non-existent in the SSC sample). Of note, the SSC sample data were contributed from multiple groups in various regions, increasing the genetic heterogeneity even more in this specific sample. In addition, we would need to consider that the true effect size may have been much smaller than 1.4, an estimate from the previous studies. We also confirmed the effect of age on stereotyped behavior (i.e., older subjects with less severe stereotyped behavior), which is consistent with previous studies [39,40]. In addition, we found gender and population effects on the RBS-R subscale scores in the SSC sample. Moreover, we did not find any effect of study site in the UIC-UF sample, which is particularly interesting because the UIC and UF samples are different in terms of recruitment and assessment protocols, geographic locations, and the rate of concurrent psychotropic medications.

## Conclusions

Our study confirmed an association between *SLC25A12 *and RRB. Genetic and phenotypic heterogeneity may account for the flip-flop phenomenon of associated alleles between our study and the Silverman study, and for the absence of association between *SLC25A12 *and ASDs in the SSC trio sample. In addition, *SLC25A12 *may not be the risk allele itself but may be in LD with a real risk allele for RRB and/or ASDs. As anticipated based on the replication design, this study did not fully tag the gene, but was designed to replicate previous findings. Therefore, we identified seven tagging *SLC25A12 *SNPs with pair-wise r^2^< 80% and MAF>10% from the International Hapmap project (http://hapmap.ncbi.nlm.nih.gov/). Future research efforts should include searching for risk alleles nearby using denser genetic markers including (but not limited to) all tagging SNPs, and dense resequencing of the interval to find genetic variants possibly more directly related to phenotype.

## Competing interests

The authors declare that they have no competing interests.

## Authors' contributions

SJK carried out sample recruitment, phenotypic data collection, data processing, genotyping and data analyses, and drafted the manuscript. RMS participated in the sample processing and genotyping. CGF participated in the sample recruitment and data processing at the UF. SJ participated in the sample recruitment and data processing at the UIC. SG participated in the sample recruitment and phenotypic data collection at the UIC. GV participated in the sample recruitment and phenotypic data collection at the UF. AMZ participated in the biomaterial processing and validation of data entry. EHC conceived of the study, participated in its design and coordination, and helped to draft the manuscript. JAB designed the data analyses plan, supervised statistical analyses and helped to draft the manuscript. All authors read and approved the final manuscript.
